# An Active Control Method for a Lower Limb Rehabilitation Robot with Human Motion Intention Recognition

**DOI:** 10.3390/s25030713

**Published:** 2025-01-24

**Authors:** Zhuangqun Song, Peng Zhao, Xueji Wu, Rong Yang, Xueshan Gao

**Affiliations:** 1 College of Mechanical and Marine Engineering, Beibu Gulf University, Qinzhou 535011, China; songzq0223@163.com (Z.S.); 15779879064@163.com (X.W.); 2 School of Mechatronical Engineering, Beijing Institute of Technology, Beijing 100081, China; zhao.p1202@gmail.com; 3 Key Laboratory of Intelligent Control and Rehabilitation Technology of the Ministry of Civil Affairs, Beijing 100176, China; wwqq346@163.com

**Keywords:** human motion intention recognition, follow-up lower extremity exoskeleton rehabilitation robot, machine learning algorithm, intelligent optimization algorithm, dual radial basis function neural network adaptive sliding mode controller

## Abstract

This study presents a method for the active control of a follow-up lower extremity exoskeleton rehabilitation robot (LEERR) based on human motion intention recognition. Initially, to effectively support body weight and compensate for the vertical movement of the human center of mass, a vision-driven follow-and-track control strategy is proposed. Subsequently, an algorithm for recognizing human motion intentions based on machine learning is proposed for human-robot collaboration tasks. A muscle–machine interface is constructed using a bi-directional long short-term memory (BiLSTM) network, which decodes multichannel surface electromyography (sEMG) signals into flexion and extension angles of the hip and knee joints in the sagittal plane. The hyperparameters of the BiLSTM network are optimized using the quantum-behaved particle swarm optimization (QPSO) algorithm, resulting in a QPSO-BiLSTM hybrid model that enables continuous real-time estimation of human motion intentions. Further, to address the uncertain nonlinear dynamics of the wearer-exoskeleton robot system, a dual radial basis function neural network adaptive sliding mode Controller (DRBFNNASMC) is designed to generate control torques, thereby enabling the precise tracking of motion trajectories generated by the muscle–machine interface. Experimental results indicate that the follow-up-assisted frame can accurately track human motion trajectories. The QPSO-BiLSTM network outperforms traditional BiLSTM and PSO-BiLSTM networks in predicting continuous lower limb motion, while the DRBFNNASMC controller demonstrates superior gait tracking performance compared to the fuzzy compensated adaptive sliding mode control (FCASMC) algorithm and the traditional proportional–integral–derivative (PID) control algorithm.

## 1. Introduction

In recent years, stroke has emerged as a major cause of death and disability among adults [1,2]. Statistics indicate that Europe has approximately 1.4 million new stroke cases annually, while the United States reports over 800,000 new cases each year [3]. In China, there are 28.76 million current and former stroke patients, with 3.94 million new strokes and 2.19 million new deaths each year [4], with the incidence rate on the rise. Among these patients, around 65% require rehabilitation [5]. Traditional manual rehabilitation training faces many challenges, including a high patient volume and a limited number of rehabilitation specialists, which makes it difficult to achieve consistent training. Additionally, the costs of manual rehabilitation training are quite high [6]. In contrast, rehabilitation robots present notable benefits, such as providing consistent repetitive training and allowing for flexible and adjustable training intensity. They can deliver repetitive rehabilitation exercises for the legs, thereby enhancing patients’ physical ability [7]. This approach aids in promoting the compensation or reorganization of neural tissue function, remedying the functional deficits of damaged nerve cells, and ultimately facilitating the recovery of neural function to help patients restore their normal gait early recovery [8,9,10].

To realize the rehabilitation goals of stroke patients, the medical field typically integrates physiotherapy techniques (like electrical stimulation and massage) with treadmill-assisted exercise. Bodyweight-supported treadmill training (BWSTT) plays a dominant role in rehabilitation training. By adjusting the level of weight support, BWSTT can reduce the load on the lower limbs, providing safe and repeatable small-range gait training [11,12]. However, BWSTT is primarily conducted on a treadmill, lacking in real environments. Long-term reliance on BWSTT may affect the recovery of natural gait. To address the issue of abnormal gait in the BWSTT system, ground-walking exoskeleton systems have been introduced into rehabilitation training. These systems are typically equipped with active hip and knee joint devices [13], assisting patients in achieving ground-based walking. However, this type of exoskeleton faces challenges in maintaining balance, requiring users to exert significant effort using crutches or handrails for support. Additionally, they need to perform hip-lifting movements during walking to compensate for the vertical movement of the center of mass, which adds extra difficulty to gait training. To enhance patients’ active participation and improve the effectiveness of rehabilitation training, introducing human motion intention recognition technology is particularly important. This method can detect the patient’s movement state during rehabilitation exercises, ensuring both the safety and effectiveness of the rehabilitation process [14]. Human motion intention can be interpreted from two levels: dynamics and kinematics. At the dynamics level, the focus is on parameters related to force/torque in the human body, such as joint torque [15] and adaptive stiffness [16]. At the kinematics level, attention is paid to movement characteristics such as joint angles [17], joint angular velocity [18], and joint angular acceleration. This study primarily focuses on the kinematics level, identifying patients’ motion intentions during rehabilitation training by analyzing parameters such as joint angles, angular velocity, and angular acceleration. This approach enables a more precise capture of the patient’s movement characteristics, allowing for a more targeted rehabilitation training plan that promotes neurological recovery and gait reconstruction.

The sEMG signals are common physiological indicators of human motion intention. Through feature extraction algorithms, information highly correlated with the wearer’s motion intention can be extracted from biological signals to predict their motion intention [19,20]. There exist two main approaches for active gait control based on sEMG signals. The first method uses sEMG signals to identify different movement patterns, with a focus on designing classification algorithms with high recognition rates and multiple movement patterns [21,22]. However, sEMG signals with a fixed number of channels can only recognize a limited range of movement patterns, and the recognition results usually serve only as trigger signals to control the robot’s movement. This may affect the coordination between the exoskeleton and patients who still retain some lower limb muscle strength. The second method continuously estimates motion variables using sEMG signals, thereby achieving smooth motion control. To address the issue of delayed motion control in exoskeletons, Wang et al. proposed a lower limb continuous motion estimation method combining sEMG signals, deep belief networks, and random forest algorithms. This method enhances the accuracy and response speed of exoskeleton robot motion control by recognizing the wearer’s motion intentions [23]. Sun et al. introduced a feature-based convolutional neural network-bi-directional long-short term memory (CNN-BiLSTM) network model that integrates sEMG and inertial measurement unit (IMU) data for more accurate and real-time knee joint angle prediction. By optimizing feature selection using ensemble feature scorer (EFS) and profile likelihood maximization (PLM) algorithms, they significantly improved prediction performance [24]. Experimental results showed that this method outperformed traditional methods in both accuracy and real-time performance, thereby rendering it appropriate for lower limb rehabilitation training. Zeng et al. proposed a sEMG–Transformer-based continuous multi-joint angle motion prediction model for the lower limbs, which was applied to a lower extremity exoskeleton rehabilitation robot, successfully achieving more precise motion intention estimation and synchronized walking [25]. Experimental results indicated that this model outperformed existing methods such as convolutional neural networks (CNNs), back propagation (BP), and long short-term memory networks (LSTMs) in prediction performance. Although the above methods have been successfully validated on specific platforms, the complex modeling and calibration processes limit their broad application across individuals. To solve this problem, future research should focus on simplifying the model calibration process and improving its adaptability and generalization across different individuals, thereby promoting the widespread application of sEMG-based active gait control in rehabilitation training.

The motion control strategies for exoskeleton robots are closely relevant to intention recognition [26]. The control algorithm should follow the user’s motion intention as soon as human movement is recognized [27], making sure the robot moves in the same direction as the human body [28,29]. In the research on control algorithms for lower limb exoskeletons, iterative learning algorithms [30], adaptive algorithms [31], and fuzzy PID algorithms [32,33] are often used in embedded development. More researchers are integrating AI techniques as a result of the rapid development of computer technology and artificial intelligence—such as neural networks [34], machine learning [35], adaptive control [36], sliding mode control [37], and intelligent swarm algorithms [38,39]—with traditional control technologies to achieve precise tracking of human gait curves by exoskeletons. A humanoid neural network sliding mode controller based on human gait was proposed by Yu et al. With parameterized gait trajectories as targets, this controller creates a humanoid model based on human biomechanics and designs a humanoid control system for the robot. Given the errors in the dynamic model of the robot, neural networks are used to improve the precision of the model by compensating for the uncertain aspects. Furthermore, the system’s sliding mode control enhances stability, tracking efficiency, and response time [40]. Sun et al. introduced a compound position control strategy that employs secondary sliding mode control. This method utilizes bis-finite-time observers to monitor and compensate for two types of perturbations in real-time, and a super-twisting approach for the position control part to guarantee that the trajectory tracking error of the knee joint converges to zero in finite time [41]. Kenas et al. employed two radial basis function (RBF) neural networks to precisely estimate perturbations and uncertainty parameters. They integrated backstepping techniques with the super-twisting algorithm to reduce tracking errors, and the controller’s stability was validated using Lyapunov theory, ensuring precise tracking of the target trajectory [42]. In summary, the combination of RBF neural networks with sliding mode control can effectively compensate for and counteract external disturbances and friction, enabling precise tracking of gait trajectories in LEERR.

Existing LEERR systems have certain limitations in design and application, resulting in low levels of patient engagement in rehabilitation training and have consequently reduced rehabilitation efficacy. To address these issues, this study focuses on the following three key questions: (1) How can we combine the advantages of body-weight-supported and follow-up lower limb exoskeleton platforms to develop a new follow-up LEERR and propose a completed active control solution? (2) How can we leverage physiological information from the human body to achieve accurate and continuous motion intention estimation, enhancing the wearer’s active participation and thereby promoting more effective rehabilitation training? (3) How can we select an appropriate trajectory tracking controller to achieve precise gait trajectory tracking control for trajectories generated at the muscle–machine interface?

## 2. Methods

### 2.1. Overall System Framework

**Figure 1:**
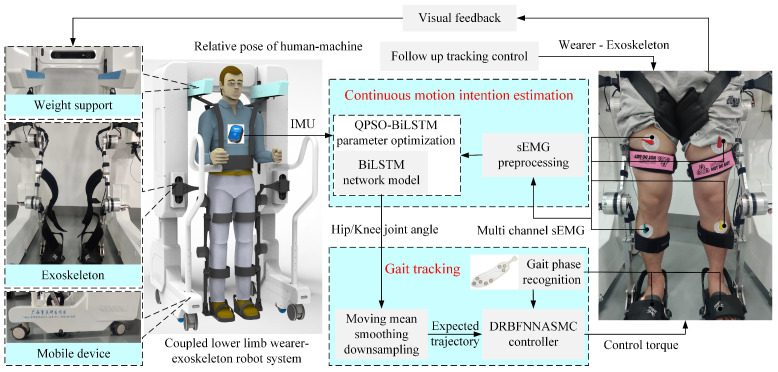
Active control framework of LEERR.

The framework of the proposed active control algorithm in this study is illustrated in Figure 1. The device mainly comprises three components: (1) Follow-up assistive frame: This component regulates the vertical motion of a weight-reducing cantilever by retracting and releasing a steel cable via a DC motor to provide follow-up body weight support. This design prevents potential falls during rehabilitation and compensates for the vertical movement of the center of mass during walking, correcting the improper hip-lifting gait post-stroke. (2) LEERR: The robot employs a bionic humanoid joint exoskeleton design to achieve high consistency between the exoskeleton and human joint movements, effectively simulating and supporting natural gait motion. (3) Sensor information system: This system includes a ZED stereo camera, IMU, and sEMG sensors. The ZED stereo camera captures depth information between the user and the follow-up assistive frame, while the IMU and sEMG sensors collect joint angles and sEMG signals to establish a mapping relationship between joint movements and myoelectric signals. The theoretical research also consists of three parts: (1) 3D human information extraction: Using the Python-OpenPose algorithm to detect the 2D shoulder information of the human body and combining it with binocular ranging principles to obtain depth information, a velocity controller is designed to achieve active tracking of the follow-up assistive frame. (2) Muscle–machine interface construction: A BiLSTM network is employed to construct the muscle–machine interface, mapping multi-channel sEMG signals in real-time to hip and knee joint movement angles. The QPSO algorithm is used for optimizing the hyperparameters of the BiLSTM network, forming a QPSO-BiLSTM model for continuous real-time estimation of human motion intentions. (3) Trajectory tracking controller design: A DRBFNNASMC controller is designed to generate control torques, enabling the robot to accurately track the trajectories generated by the muscle–machine interface.

### 2.2. Vision-Driven Follow-Up Tracking Control

In this section, the Python-OpenPose algorithm is used for detecting the 2D message of the human shoulder, which is then combined with binocular vision to obtain depth information, enabling the perception of the relative 3D coordinates of the human during walking. Based on this information, a kinematic model of the follow-up assistive frame is established, and a velocity controller is designed to actively follow human motion.

#### 2.2.1. Three-Dimensional Human Information Extraction Based on Python-OpenPose and Binocular Vision

In the process of achieving active tracking using the assistive frame, perceiving the relative 3D coordinate information of a human during walking is essential. Many researchers have conducted in-depth studies on this content. Hou et al. proposed a feature-matching method combining the scale-invariant feature transform and random sample consensus algorithms to provide 2D navigation information for mobile robots, achieving precise path tracking and navigation [43]. Quan et al. introduced a navigation algorithm that integrates deep reinforcement learning and recurrent neural networks. By optimizing path exploration with dual networks and recurrent neural modules, they improved pathfinding efficiency and reduced path length [44]. Efstathiou et al. presented a lightweight framework that combines CNNs with LSTM networks for detecting and identifying gait features from 2D laser sensor data, enhancing the accuracy and efficiency of online leg tracking and gait analysis for mobile assistive robots [45]. Wang et al. introduced a visual navigation approach that utilizes object-level topological semantic mapping in conjunction with heuristic search techniques. This methodology employs active visual perception and goal-oriented path segmentation, resulting in the generation of smooth trajectories through the application of Bernstein polynomials, thereby improving the navigation success rate and path efficiency of autonomous mobile robots [46].

Although the aforementioned methods can successfully detect human-related information, using multiple sensors to obtain navigation information is costly. Binocular vision, as a single sensor source, offers high robustness and completeness but relies on precise binocular feature matching. OpenPose, an open-source human pose estimation library, can accurately identify human skeletal points in 2D and 3D spaces, providing precise joint positions for improved tracking of human relative position and posture. Therefore, this study captures human motion posture using a ZED stereo camera, detects the 2D information of the human shoulder through the Python-OpenPose algorithm, and obtains depth information with binocular vision, thus avoiding the complexity of binocular feature matching.

**Figure 2:**
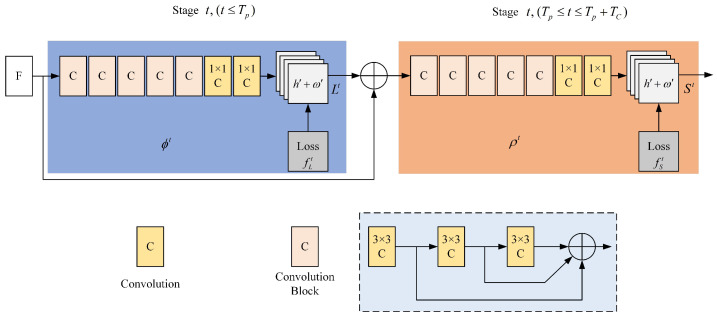
OpenPose network structure.

The network structure of OpenPose, illustrated in Figure 2, is primarily divided into two components: the left side is used for predicting the affinity field of key points, whereas the right side is used for predicting the confidence of key points. The input image is processed through a 10-layer Visual Geometry Group 19-layer network (VGG-19) network to extract image features *F*. Which are then used to generate predictions for the part affinity fields (PAF) and part confidence maps (PCM), respectively. Figure 2, $\phi^{t}$ and $\rho^{t}$ represent the CNNs used for predicting the PAF and PCM. The prediction formulas for the vector field and confidence are as follows:

(1) $$\begin{array}{c} L^{1}=\phi^{1}\left(F\right),\;t=1\\ L^{t}=\phi^{t}\left(F,L^{t-1}\right),\;\forall 2\leq t\leq T_{p}\\ S^{T_{p}+1}=\rho^{t}\left(F,L^{T_{p}}\right),\;t=T_{p}+1\\ S^{t}=\rho^{t}\left(F,L^{T_{p}},S^{t-1}\right),\;\forall T_{p}+2\leq t\leq T_{p}+T_{C} \end{array}$$

The network specifies the subsequent loss function for each stage *t* in each layer:

(2) $$\begin{array}{c} f_{L}^{t}=\sum_{c=1}^{C}\sum_{P}W(P)\cdot {∥L_{c}^{t}\left(P\right)-L_{c}^{*}\left(P\right)∥}_{2}^{2}\\ f_{S}^{t}=\sum_{j=1}^{J}\sum_{P}W(P)\cdot {∥S_{j}^{t}\left(P\right)-S_{j}^{*}\left(P\right)∥}_{2}^{2}\\ f=\sum_{t=1}^{T}\left(f_{L}^{t}+f_{S}^{t}\right) \end{array}$$

where W(P) represents a binary bit, when W(P)=0 indicates that the position of point *P* is not labeled. $L_{c}^{t}\left(P\right)$ and $S_{j}^{t}\left(P\right)$ denote the network’s predicted value at point *P*, and $L_{c}^{*}\left(P\right)$ and $S_{j}^{*}\left(P\right)$ refer to the network’s actual values at point *P*. While training the confidence for key points, the confidence level for key points $p_{j}$ of the *k*th individual in the image exhibits akin to a Gaussian distribution centered around that position.

(3) $$\begin{array}{c} S_{j,k}^{*}\left(P\right)=\exp\left(-\frac{{∥P-x_{j,k}∥}_{2}^{2}}{\sigma^{2}}\right)\\ S_{j}^{*}\left(P\right)=\max_{k}S_{j,k}^{*}\left(P\right) \end{array}$$

where *k* represents the *k*th individual, σ signifies the variance in the Gaussian function that regulates the range of actual joints. A higher value σ leads to an increase in $S_{j,k}^{*}\left(P\right)$, which in turn results in a decrease in prediction accuracy.

**Figure 3:**
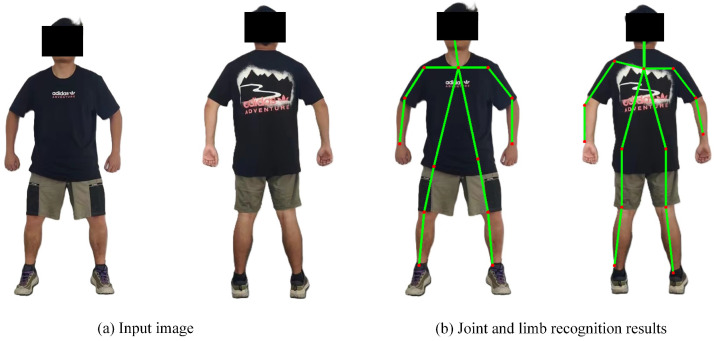
Actual results of human joint and limb recognition based on Python-OpenPose. (The green lines represent the main skeleton of the human body, and the red dots represent the main key points of the human body).

For the limb *c* of the *k*th individual in the image, the affinity vector field defined at pixel point *P* is as follows:

(4) $$\begin{array}{c} L_{c,k}^{*}\left(P\right)=\left\{\begin{array}{cc} v & if\;P\;on\;\lim b\;c\;of\;the\;kth\;person\\ 0 & otherwise \end{array}\right.\\ L_{c}^{*}\left(P\right)=\frac{1}{n_{c}\left(P\right)}\sum_{k}L_{c,k}^{*}(P) \end{array}$$

where $v=\left(x_{j2,k}-x_{j1,k}\right)/{∥x_{j2,k}-x_{j1,k}∥}_{2}$ is the unit vector that indicates the direction of the extremity, whereas $x_{j1,k}$ and $x_{j2,k}$ are the actual positions of $j_{1}$ and $j_{2}$. When there is an overlap in the image, the average value of the affinity vector field at that point should be taken. By extracting the key points and their affinity vector fields, clustering analysis and optimization of the key points are performed, ultimately constructing the human skeleton(s) for single or multiple persons in the image. Figure 3 illustrates the outcomes of extracting the actual human skeleton using OpenPose, with the left and right corresponding to the input and output images of the ZED stereo camera, respectively, directly obtaining the 2D information of the main human joints. Considering the recognition accuracy of OpenPose and the placement of the ZED stereo camera on the follow-up assistive frame, this study primarily extracts the coordinates of both shoulders to guide tracking information for the follow-up assistive frame.

**Figure 4:**
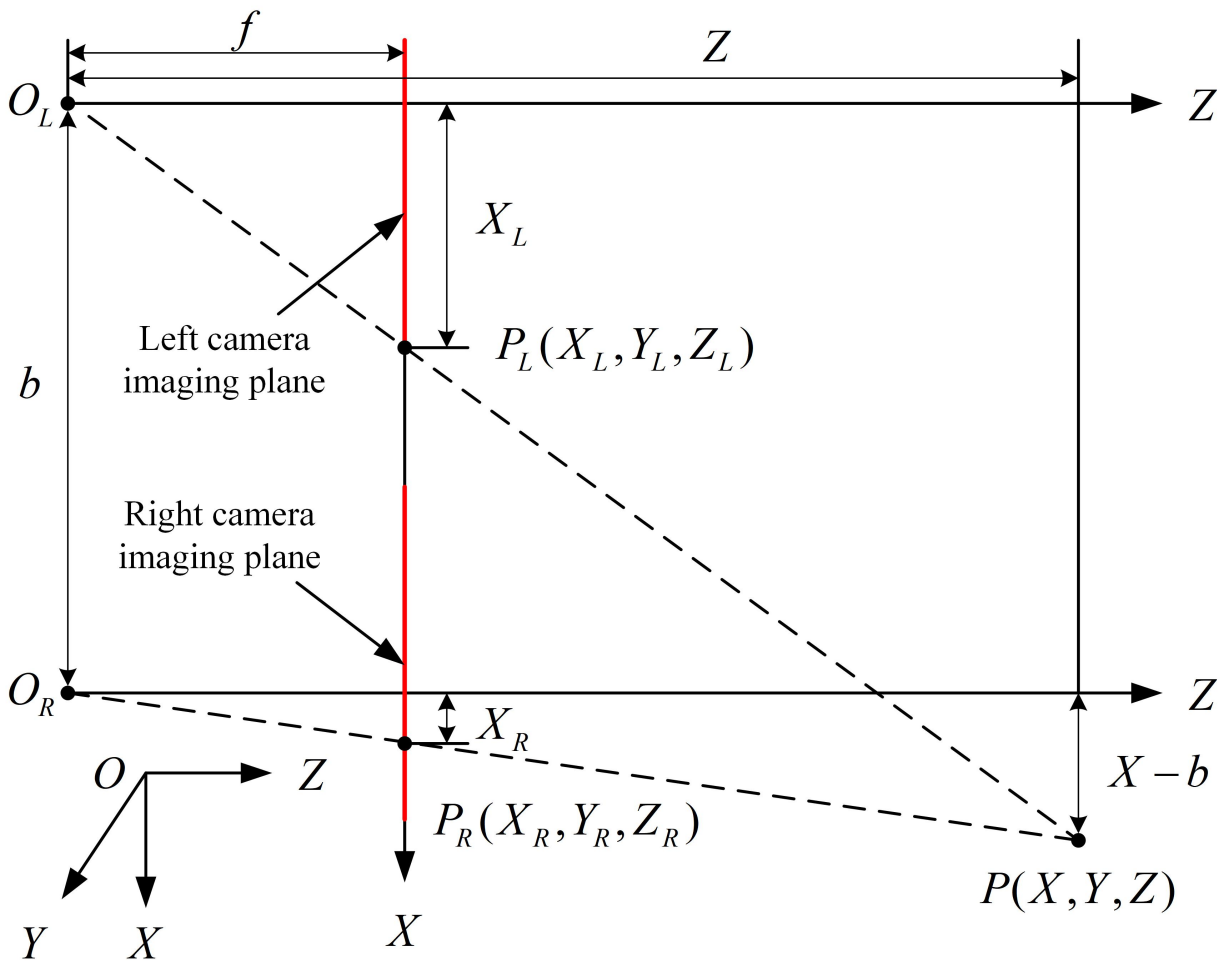
Principle of binocular ranging.

The above process only obtains the 2D shoulder coordinates of the human body, lacking depth information between the person and the camera. Since this study uses a stereo camera, depth information can be extracted using the principle of binocular vision. The principle of binocular vision is shown in Figure 4, where *P* represents the human shoulder. $O_{L}$ and $O_{R}$ are the optical centers of both cameras, whereas $P_{L}$ and $P_{R}$ are the image points on both sides. $X_{L}$ and $X_{R}$ denote the distances from the left and right image points to their corresponding optical centers, *f* is the focal length of the camera, *b* is the baseline distance of both camera centers, and *Z* is the depth information of *P* relative to optical centers. Based on the principle of similar triangles, the following relationship can be derived: $Z=\frac{fb}{X_{L}-X_{R}}$. Thus, once the disparity $X_{L}-X_{R}$ is obtained, the depth information of the shoulder coordinates can be determined. Through the combination of Python-OpenPose and binocular vision, the 3D coordinates of both shoulders relative to the binocular center of the ZED stereo camera can be obtained, with the left shoulder coordinates denoted as $P_{L}\left(X_{L},Y_{L},Z_{L}\right)$ and the right shoulder coordinates as $P_{R}\left(X_{R},Y_{R},Z_{R}\right)$.

#### 2.2.2. Follow-Up Assistive Frame Tracking Control

**Figure 5:**
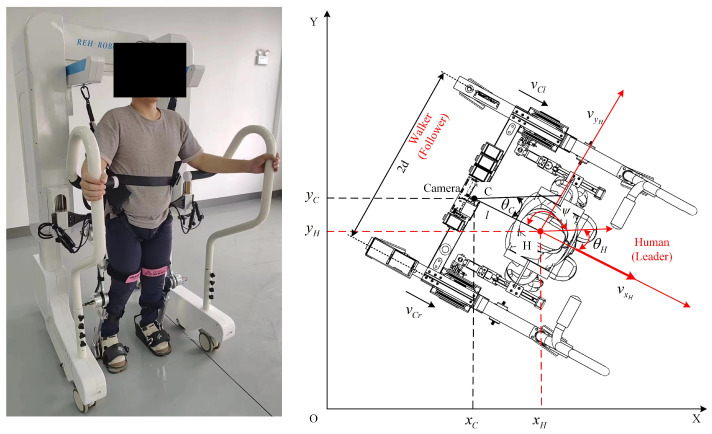
Human follow-up-assisted frame kinematic model.

The kinematic model of the follow-up assistive frame is shown in Figure 5. The pose of the assistive frame in the coordinate system OXY is represented by the vector ${\left(x_{C},y_{C},\theta_{C}\right)}^{T}$, where $x_{C}$ and $y_{C}$ are the horizontal and vertical coordinates of the assistive frame in the *X*-*Y* plane. In this study, the coordinates of the assistive frame are set at the ZED stereo camera. The distance between the two drive wheels is 2d, the radius of each drive wheel is *r*. $\theta_{C}$ is the orientation angle of the assistive frame, whereas $v_{C}$ and $\omega_{C}$ represent the linear and angular velocities of the assistive frame, respectively. In practical use, the assistive frame is equipped with anti-slip wheels, preventing lateral movement. Additionally, assuming that the centroid coincides with the geometric center, the frame must satisfy the pure rolling and no-slip conditions. Therefore, the following constraint equations apply:

(5) $$\begin{array}{c} {\dot{x}}_{C}\sin\theta_{C}-{\dot{y}}_{C}\cos\theta_{C}=0\\ {\dot{x}}_{C}\cos\theta_{C}+{\dot{y}}_{C}\sin\theta_{C}+d{\dot{\theta }}_{C}=v_{Cr}\\ {\dot{x}}_{C}\cos\theta_{C}+{\dot{y}}_{C}\sin\theta_{C}-d{\dot{\theta }}_{C}=v_{Cl} \end{array}$$

The kinematic equations for the differential-drive follow-up assistive frame with two wheels are as follows:

(6) $${\dot{q}}_{C}=\left[\begin{array}{c} {\dot{x}}_{C}\\ {\dot{y}}_{C}\\ {\dot{\theta }}_{C} \end{array}\right]=\left[\begin{array}{cc} \cos\theta_{C} & 0\\ \sin\theta_{C} & 0\\ 0 & 1 \end{array}\right]\left[\begin{array}{c} v_{C}\\ \omega_{C} \end{array}\right]$$

During actual movement, the coordinates of the assistive frame can be obtained through encoders on the two drive wheels, and the coordinates of the person can be calculated using feedback parameters from the assistive frame and camera. The coordinates of the person can be represented as follows:

(7) $$\begin{array}{c} x_{H}=x_{C}-l\cos\psi \\ y_{H}=y_{C}-l\sin\psi \end{array}$$

where *l* is the distance between the person and the camera, given by $l=\frac{Z_{R}+Z_{L}}{2}$. ψ is the orientation angle of the line connecting the person and the assistive frame. Since the assistive frame continuously moves in the direction of the person’s motion and the person holds the handrails on either side of the frame during movement, this geometric relationship can be approximated as $\theta_{C}+\psi =\pi$.

During movement, the assistive frame needs to maintain a certain desired state relative to the person. Let the desired distance be $l_{d}$ and the desired orientation angle be $\psi^{d}$. To achieve active following, the desired state of the assistive frame is defined by the person’s coordinates:

(8) $$\begin{array}{c} x_{C}^{d}=x_{H}-l^{d}\cos\psi^{d}\\ y_{C}^{d}=y_{H}-l^{d}\sin\psi^{d}\\ \theta_{C}^{d}=\theta_{H} \end{array}$$

The tracking error of the assistive frame is defined as follows:

(9) $$e_{\theta C}=\left[\begin{array}{c} x_{eC}\\ y_{eC}\\ \theta_{eC} \end{array}\right]=\left[\begin{array}{ccc} \cos\theta_{C} & \sin\theta_{C} & 0\\ -\sin\theta_{C} & \cos\theta_{C} & 0\\ 0 & 0 & 1 \end{array}\right]\left[\begin{array}{c} x_{C}^{d}-x_{C}\\ y_{C}^{d}-y_{C}\\ \theta_{C}^{d}-\theta_{C} \end{array}\right]$$

where $\theta_{eC}$ can be fed back in real-time through the geometric relationship $\theta_{eC}=\arctan\frac{Y_{R}-Y_{L}}{X_{R}-X_{L}}$ in double-shoulder coordinates. Consequently, the error differential matrix for the assistive frame is as follows:

(10) $${\dot{e}}_{\theta C}=\left[\begin{array}{c} {\dot{x}}_{eC}\\ {\dot{y}}_{eC}\\ {\dot{\theta }}_{eC} \end{array}\right]=\left[\begin{array}{c} \omega_{C}y_{eC}-v_{C}+v_{C}^{d}\cos\theta_{eC}\\ -\omega_{C}x_{eC}+v_{C}^{d}\sin\theta_{eC}\\ \omega_{C}^{d}-\omega_{C} \end{array}\right]$$

A globally asymptotically stable velocity tracking controller has been designed according to the differential equations of the postural error, along with the method of inverse and Lyapunov stability theory. This controller utilizes the output linear velocity $v_{C}$ and angular velocity $\omega_{C}$ as assist control inputs to design the velocity controller for the follow-up-assisted frame:

(11) $$\left[\begin{array}{c} v_{C}\\ \omega_{C} \end{array}\right]=\left[\begin{array}{c} v_{C}^{d}\cos\theta_{eC}+k_{1}x_{eC}\\ \omega_{C}^{d}+k_{2}\theta_{eC}+k_{3}v_{C}^{d}\sin\theta_{eC} \end{array}\right]$$

where $k_{1}$, $k_{2}$ and $k_{3}$ are all positive constants, and $v_{C}^{d}$ and $\omega_{C}^{d}$ are the desired linear and angular velocities, respectively. It can be obtained by taking the derivative of Equation (8) with respect to time.

### 2.3. Human Motion Intention Recognition

The methods for recognizing human motion intentions are typically categorized into discrete action classification [47] and continuous motion estimation [23,24,25]. Discrete action classification is limited to recognizing combinations of discrete joint movements and does not facilitate the continuous and unrestricted motion of a robot’s joints like human joints. In contrast, continuous motion estimation allows for smooth and continuous control of the robot’s joints by the patient, thus improving the coordination between stroke patients with partial autonomous movement ability and exoskeleton robots. From a kinematic perspective, human motion intention can be analyzed as joint angle trajectories, allowing for the establishment of a correlation between sEMG signals and joint angles. However, there is a complex nonlinear correlation exists between sEMG signals and joint angles. In this study, a BiLSTM network is used to build a muscle–machine interface that maps multi-channel sEMG signals into the motion angles of the lower limb joint in real time. Furthermore, the QPSO algorithm is used for optimizing the hyperparameters of the BiLSTM network, forming a QPSO-BiLSTM combined model to achieve continuous and real-time estimation of human motion intentions.

#### 2.3.1. Quantum-Behaved Particle Swarm Optimization

QPSO is an advanced optimization algorithm that combines the concepts of quantum computing with traditional particle swarm optimization (PSO). In this algorithm, the state of a particle is determined by a wave function, meaning that the probability of a particle appearing in the search space depends solely on the amplitude of the wave function, unaffected by its previous trajectory. This characteristic enables particles to search the entire feasible space, increasing search diversity and helping to avoid local optima.

In QPSO, particles are updated through observation to obtain new individuals. Specifically, given the probability of observing a particle, its position can be determined. For each particle, multiple probabilities are randomly generated, and Monte Carlo methods are used to observe and obtain multiple individuals. The best individual among them is then selected, with the remaining individuals evaluated sequentially to ultimately obtain the next generation.

In QPSO, the average value mBest is introduced to denote the historical best location of the particle swarm pBest. The update steps for particles in the QPSO algorithm are as follows:

Step 1: Calculate the average best position mBest [48].

(12) $$mBest=\frac{1}{M}\sum_{i=1}^{M}pBest_{i}$$

where *M* represents the total count of particles, and $pBest_{i}$ indicates the pBest of the *i*th particle in the iteration.

Step 2: Update the particle positions.

(13) $$p_{i}=\varphi \cdot pBest_{i}+\left(1-\varphi \right)\cdot gBest$$

where φ is a random number between $\left(0,1\right)$, gBest denotes the current globally optimal particle, and $p_{i}$ is used to update the location of the *i*th particle.

The equation for updating the location of a particle is as follows:

(14) $$X_{i}\left(t+1\right)=\left\{\begin{array}{c} p_{i}+\beta \cdot \left|m_{best}\left(t\right)-X_{i}\left(t\right)\right|\cdot \ln\left(\frac{1}{u}\right),\;\;0.5\leq \psi \leq 1\\ p_{i}-\beta \cdot \left|m_{best}\left(t\right)-X_{i}\left(t\right)\right|\cdot \ln\left(\frac{1}{u}\right),\;\;0\leq \psi <0.5 \end{array}\right.$$

where ψ and *u* are random numbers between $\left(0,1\right)$, taken with a probability of ±0.5. $X_{i}\left(t+1\right)$ represents the location of the *i*th particle, while β denotes the contraction–expansion coefficient of the QPSO, which is used to control the rate of convergence. The most common way to adjust β is to change linearly, and this is generally performed according to the following equation:

(15) $$\beta =\beta_{\max}-\left(\beta_{\max}-\beta_{\min}\right)\cdot \frac{t}{t_{\max}}$$

where $t_{\max}$ denotes the max number of iterations, and $\beta_{\max}$ and $\beta_{\min}$ represent the max and min values of the parameter β, respectively. Typically, β≤1.

#### 2.3.2. BiLSTM Network

**Figure 6:**
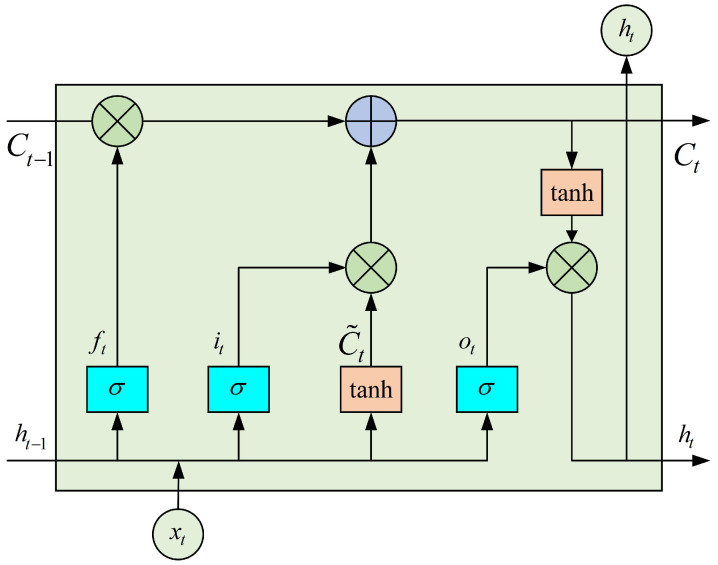
LSTM unit structure.

To address the issue of long-term dependency in recurrent neural networks (RNNs), researchers have proposed various new recurrent network architectures, such as LSTM networks, gated recursive units (GRUs) and BiLSTM networks. The LSTM network, by changing the recurrent structure of the RNN model, can effectively handle long-sequence data and offers enhanced long-term memory and modeling capabilities [49]. The architecture of LSTM is illustrated in Figure 6.

Unlike traditional neural networks, the key parts of LSTM are the unit state and various gating mechanisms, which mainly include the forgetting gate, input gate, and output gate. Together, these determine the update of the unit state. These gating mechanisms use the sigmoid function to control the degree of information flow.

(1) Forget Gate: The first step in LSTM is to decide, via the forget gate, what information should be discarded from the unit state. It also extracts useful information from the external inputs $x_{t}$ and $h_{t-1}$ to incorporate into the internal state.

(16) $$f_{t}=\sigma \left(W_{f}\cdot \left[h_{t-1},x_{t}\right]+b_{f}\right)$$

where $W_{f}$ and $b_{f}$ represent the weights and biases associated with the forgetting gate, respectively, while σ denotes the sigmoid function.

(2) Input Gate: The input gate is made up of two portions: a sigmoid layer that confirms which information will be renewed, and a tan *h* layer that produces a candidate vector containing the new information to be added.

(17) $$i_{t}=\sigma \left(W_{i}\cdot \left[h_{t-1},x_{t}\right]+b_{i}\right)$$

(18) $${\tilde{C}}_{t}=\tan h\left(W_{c}\cdot \left[h_{t-1},x_{t}\right]+b_{c}\right)$$

where $W_{i}$ and $W_{c}$ represent the weights of the input gate and input node, respectively, whereas $b_{i}$ and $b_{c}$ denote the deviations, respectively.

By combining Equtions (16)–(18) the state information of the memory cell $C_{t}$ can be obtained, which updates by forgetting the part unit and renewing the memory unit.

(19) $$C_{t}=C_{t-1}\cdot f_{t}+{\tilde{C}}_{t}\cdot i_{t}$$

(3) Output Gate: The output gate determines the portion of the unit state that will be utilized for generating the output.

(20) $$o_{t}=\sigma \left(W_{o}\cdot \left[h_{t-1},x_{t}\right]+b_{o}\right)$$

where $W_{o}$ and $b_{o}$ are the output gate weights and biases, respectively.

In conclusion, the unit state undergoes processing through a tan *h*, which transforms the values to a range of −1∼1. Subsequently, this modified cell state is multiplied by the output from the output gate, thereby ensuring that only the designated cell state is produced as output.

(21) $$h_{t}=o_{t}\cdot \tan h\left(C_{t}\right)$$

where $C_{t}$ is the unit state and $o_{t}$ is the output gate.

Although LSTM addresses the issues of gradient vanishing or explosion in traditional RNNs [50], conventional LSTM networks can only encode information from one direction, meaning they process time-series data sequentially from the past to the future. In the LSTM model, exoskeleton gait trajectory parameters are trained as a time series from front to back. This unidirectional training method results in lower data utilization and fails to fully exploit intrinsic data features. Compared to traditional unidirectional LSTM networks, the BiLSTM network consists of two LSTM layers, forward and backward, which capture both past and future state information, thus improving prediction accuracy. By building a bidirectional RNN, BiLSTM effectively overcomes the limitations of LSTM in data information utilization [51,52].

**Figure 7:**
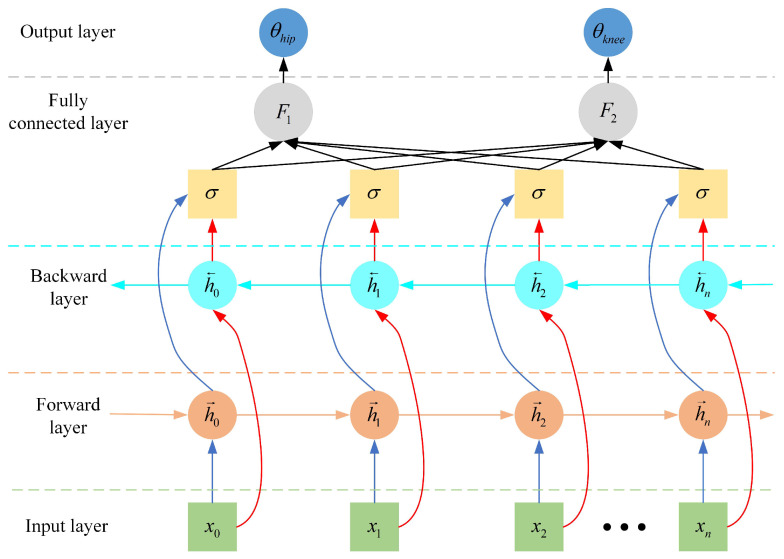
BiLSTM model structure.

Therefore, we use a BiLSTM network to construct a muscle–machine interface to predict the lower limb joint angles. The structure of the BiLSTM model is shown in Figure 7, consisting mainly of an input layer, two LSTM layers (forward and backward), a fully connected layer, a forgetting layer, and an output layer. The input layer has six nodes, corresponding to the processed six-channel sEMG sequence signals. The fully connected layer integrates data features and performs classification, outputting results for two categories. The output layer consists of two nodes, corresponding to the flexion/extension joint angle values of the hip and knee joints. Through BiLSTM network training, a mapping between multi-channel sEMG signals and joint angles is achieved, generating the desired trajectories of the lower limb joints $y_{i}^{\prime }\left(t\right)\in R^{2}$.

(22) $$\begin{array}{c} {\vec{h}}_{t}=LSTM\left(x_{t},{\vec{h}}_{t-1}\right)\\ {\overset{\leftarrow }{\;h}}_{t}=LSTM\left(x_{t},{\overset{\leftarrow }{h}}_{t-1}\right)\\ {\;h}_{t}=\vec{W}{\vec{h}}_{t}+\overset{\leftarrow }{W}{\overset{\leftarrow }{h}}_{t} \end{array}$$

where ${\vec{h}}_{t}$ and ${\overset{\leftarrow }{\;h}}_{t}$ represent the forward and backward LSTM layers, respectively, whereas $\vec{W}$ and $\overset{\leftarrow }{W}$ denote the weight coefficients, respectively.

#### 2.3.3. QPSO-BiLSTM Gait Trajectory Prediction

The QPSO algorithm is used for optimizing the parameters of the BiLSTM network, specifically the number of iterations, neurons, and the learning rate. After reading the time series, the dataset is partitioned into a training set and a test set, adhering to a 9:1 ratio. Once the BiLSTM network structure is determined, the neural network parameters are initialized, and QPSO optimizes the randomly initialized particle positions. The dataset is used to calculate the fitness value of each individual, which serves as one of the evaluation criteria. If the QPSO training criteria are met, the optimized parameters, such as the learning rate, are input into the BiLSTM network for training. Subsequently, the network undergoes validation utilizing the test set, resulting in the generation of predicted values. Following this, an anti-normalization is applied to obtain the ultima results. Eventually, the gait trajectory prediction performance is evaluated using mean absolute error ($\delta_{MAE}$), mean absolute percentage error ($\delta_{MAPE}$), root mean square error ($\delta_{RMSE}$), and coefficient of determination ($R^{2}$). The equations are delineated as follows:

(23) $$\delta_{MAE}=\frac{1}{n}\sum_{i=1}^{n}\left|y_{i}-{y^{\prime }}_{i}\right|$$

(24) δMAPE=100%n∑i=1nyi−y′iyi−y′iyiyi

(25) $$\delta_{RMSE}=\sqrt{\frac{1}{n}{\sum_{i=1}^{n}\left(y_{i}-{y^{\prime }}_{i}\right)}^{2}}$$

(26) $$R^{2}=1-\frac{\sum_{i=1}^{n}{\left(y_{i}-{y^{\prime }}_{i}\right)}^{2}}{\sum_{i=1}^{n}{\left(y_{i}-{\bar{y}}_{i}\right)}^{2}}$$

where *n* stands for the number of predictions, $y_{i}$ refers to the real gait trajectory values, $y_{i}^{\prime }$ indicates the estimated values of gait trajectory, and ${\bar{y}}_{i}$ represents the mean of the true values.

**Figure 8:**
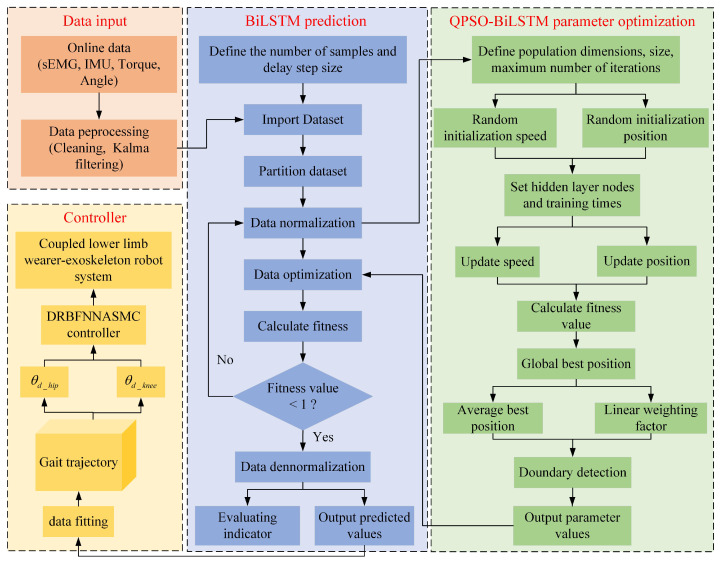
QPSO-BiLSTM gait trajectory prediction framework.

The gait trajectory prediction process includes four parts: data input, prediction, optimization, and control, as illustrated in Figure 8. The detailed steps are outlined below:

Step 1: The collected online data, including IMU and sEMG signals, are used as the model’s input.

Step 2: The sample data undergo normalization to a range of $\left[0,1\right]$ through the application of min–max normalization, according to Equation (27). Subsequently, the dataset is partitioned into training and testing samples in a ratio of 9:1.

(27) $$x^{\prime }=\frac{x-x_{\min}}{x_{\max}-x_{\min}}$$

where $x^{\prime }$ is the normalized data, whereas $x_{\min}$ and $x_{\max}$ are the min–max values of the sample, and *x* is the original sample data.

Step 3: Initialize the model’s hyperparameters, setting the number of hidden layers to *m*, learning rate to *r*, maximum iterations to $t_{\max}$, and population size to *n*.

Step 4: After randomly initializing the velocity and position, optimize the quantity of hidden layer nodes as well as the number of training iterations. Update the velocity and position, then calculate the fitness value and find the global optimal position.

Step 5: According to the global optimal position, introduce a linear weighting factor, calculate the average best position, perform boundary checking, and input it into the BiLSTM optimization module to recalculate particle fitness. If the fitness value is ≥1, recalculate; otherwise, proceed with metric evaluation and output the prediction results.

Step 6: Fit the predicted output values to obtain the hip and knee gait trajectories. Run the controller and apply it to the wearer–exoskeleton robot system.

### 2.4. Control of Trajectory Tracking for LEERR Based on Motion Intent

This section designs the DRBFNNASMC controller for tracking the trajectories generated by the muscle–machine interface. The controller achieves global asymptotic tracking of joint trajectories in the presence of friction and external perturbation, demonstrating excellent control performance. The following will provide a detailed description of the controller design process and analyze the system’s state stability under the controller’s operation.

The 2-DOF LEERR dynamics model can be described as follows:

(28) $$M\left(\theta \right)\ddot{\theta }+C\left(\theta ,\dot{\theta }\right)\dot{\theta }+G\left(\theta \right)+\tau_{f}+\tau_{dis}=\tau$$

where M(θ) represents the inertia matrix, $C(\theta ,\dot{\theta })$ denotes the centrifugal and Coriolis force matrix, G(θ) stands for the gravity matrix, $\tau_{f}$ indicates the frictional force, $\tau_{dis}$ refers to the external disturbances, and τ is the joint control torque input vector.

In actual rehabilitation training, uncertainties such as the friction between the exoskeleton insole and the ground and external disturbances are challenging to measure accurately. The RBF neural network is frequently employed for its superior approximation capabilities, particularly in modeling the nonlinear dynamics of controlled systems and minimizing approximation errors [53]. Therefore, this study adopts a dual RBF neural network to estimate the frictional force and external disturbances in Equation (25). Furthermore, by integrating the dual RBF neural network with sliding mode control, we propose the DRBFNNASMC control algorithm to track the gait trajectory predicted by the QPSO-BiLSTM. In a conventional RBF neural network, the correlation between the input and output vectors can be expressed as follows:

(29) $$h_{j}=\exp\left(-\frac{{∥x-c_{j}∥}^{2}}{2\sigma_{j}^{2}}\right)$$

(30) $$\tau_{f}\left(x\right)={\omega_{f}}^{*T}h_{f}\left(x\right)+\varepsilon_{f}$$

(31) $$\tau_{dis}\left(x\right)={v_{dis}}^{*T}h_{dis}\left(x\right)+\varepsilon_{dis}$$

where *x* is the input of the neural network, *j* is the hidden layer, $h_{j}$ is the output of the Gaussian function, $c_{j}$ is the center vector of the Gaussian function, and $\sigma_{j}$ is the width of the Gaussian function. ${\omega_{f}}^{*}$ and ${v_{dis}}^{*}$ are the ideal weights, whereas $\varepsilon_{f}$ and $\varepsilon_{dis}$ are the evaluated errors, and $\left|\varepsilon_{f}\right|\leq \varepsilon_{Nf}$, $\left|\varepsilon_{dis}\right|\leq \varepsilon_{Ndis}$. $\tau_{f}$ and $\tau_{dis}$ are the ideal outputs.

Let the input to the neural network be defined as $x={\left[e\;\dot{e}\right]}^{T}$; then, the output is given by the following:

(32) $${\hat{\tau }}_{f}\left(x\right)={\hat{\omega }}^{T}h_{f}\left(x\right),\;{\hat{\tau }}_{dis}\left(x\right)={\hat{v}}^{T}h_{dis}\left(x\right)$$

where $h_{f}\left(x\right)$ and $h_{dis}\left(x\right)$ are the Gaussian functions of the RBF neural network. For the RBF neural network, the gradient descent algorithm can be used to update the weights. The basic idea is to continuously adjust the values of ω and *v* in the direction of the negative gradient of E(x) until E(x) reaches a minimum. The approximation error criterion function for the network is defined as follows:

(33) $$E\left(x\right)=\frac{1}{2}\sum_{i=1}^{n}\sum_{j=1}^{m}{\left(y\left(x\right)-y_{m}\left(x\right)\right)}^{2}$$

where y(x) represents the desired output vector, and $y_{m}\left(x\right)$ denotes the real output vector. Following the gradient descent algorithm, the weight parameters can be modified in the following manner:

(34) $$\begin{array}{c} \Delta \omega_{j}\left(x\right)=-\eta \frac{\partial E}{\partial \omega_{j}}=\eta \left(y\left(x\right)-y_{m}\left(x\right)\right)h_{f}\\ \Delta v_{j}\left(x\right)=-\eta \frac{\partial E}{\partial v_{j}}=\eta \left(y\left(x\right)-y_{m}\left(x\right)\right)h_{dis} \end{array}$$

(35) $$\begin{array}{c} \omega_{j}\left(x\right)=\omega_{j}\left(x-1\right)+\Delta \omega_{j}\left(x\right)+\alpha \left(\omega_{j}\left(x-1\right)\right)-\omega_{j}\left(x-2\right)\\ v_{j}\left(x\right)=v_{j}\left(x-1\right)+\Delta v_{j}\left(x\right)+\alpha \left(v_{j}\left(x-1\right)\right)-v_{j}\left(x-2\right) \end{array}$$

where j=1,2,…,m. η∈(0,1) represents the learning rate, and α∈(0,1) denotes the momentum factor. As the RBF neural network learns online, E(x)→0, the weights ω and *v* will eventually stabilize, leading the system to achieve stability. The control objective for trajectory tracking is to make the actual angle value approach the ideal angle value continuously, i.e., $\theta \to \theta_{d}$, while $\theta_{d}$ is the ideal angle value, and θ is the real angle value.

Define the angle tracking error as $e=\theta -\theta_{d}$. Taking the derivative of the error obtained $\dot{e}=\dot{\theta }-{\dot{\theta }}_{d}$, the sliding mode function is defined as follows:

(36) $$s=\dot{e}+\Lambda e,\;\Lambda >0$$

Taking the derivative of Equation (36), we obtain the following:

(37) $$\begin{array}{c} \dot{s}=\ddot{e}+\Lambda \dot{e}=\ddot{\theta }-{\ddot{\theta }}_{d}+\Lambda \dot{e}\\ =M^{-1}\left(\theta \right)\left[\tau -\tau_{dis}-\tau_{f}-G\left(\theta \right)-C\left(\theta ,\dot{\theta }\right)\dot{\theta }\right]-{\ddot{\theta }}_{d}+\Lambda \dot{e} \end{array}$$

The design of the trajectory tracking controller is as follows:

(38) $$\tau =M\left(\theta \right)\left({\ddot{\theta }}_{d}-\Lambda \dot{e}-\eta \mathrm{sgn}\left(s\right)+{\tilde{\tau }}_{f}+{\tilde{\tau }}_{dis}\right)+C\left(\theta ,\dot{\theta }\right)\dot{\theta }+G\left(\theta \right)+\tau_{f}+\tau_{dis}$$

where $\tilde{\omega }=\hat{\omega }-\omega^{*},\;\tilde{v}=\hat{v}-v^{*},$ and

(39) $$\begin{array}{c} {\tilde{\tau }}_{f}\left(x\right)={\hat{\tau }}_{f}\left(x\right)-\tau_{f}\left(x\right)={\hat{\omega }}^{T}h_{f}\left(x\right)-{\omega_{f}}^{*T}h_{f}\left(x\right)-\varepsilon_{f}={\tilde{\omega }}_{f}^{T}h_{f}\left(x\right)-\varepsilon_{f}\\ {\tilde{\tau }}_{dis}\left(x\right)={\hat{\tau }}_{dis}\left(x\right)-\tau_{dis}\left(x\right)={\hat{v}}^{T}h_{dis}\left(x\right)-{v_{dis}}^{*T}h_{dis}\left(x\right)-\varepsilon_{dis}={\tilde{v}}_{dis}^{T}h_{dis}\left(x\right)-\varepsilon_{dis} \end{array}$$

Define the Lyapunov function for the closed-loop system as follows:

(40) $$L=\frac{1}{2}s^{T}s+\frac{1}{2\lambda_{1}}{\tilde{\omega }}_{f}^{T}{\tilde{\omega }}_{f}+\frac{1}{2\lambda_{2}}{\tilde{v}}_{dis}^{T}{\tilde{v}}_{dis}$$

where $\lambda_{1}>0,\lambda_{2}>0$.

Taking the derivative of Equation (40) and merging Equation (37) and Equation (38), we obtain

(41) $$\begin{array}{c} \dot{L}=s^{T}\dot{s}+\frac{1}{\lambda_{1}}{\tilde{\omega }}_{f}^{T}{\dot{\hat{\omega }}}_{f}+\frac{1}{\lambda_{2}}{\tilde{v}}_{dis}^{T}{\dot{\hat{v}}}_{dis}\\ =s^{T}\left[{\tilde{\omega }}_{f}^{T}h_{f}\left(x\right)-\varepsilon_{f}+{\tilde{v}}_{dis}^{T}h_{dis}\left(x\right)-\varepsilon_{dis}-\eta \mathrm{sgn}\left(s\right)\right]+\frac{1}{\lambda_{1}}{\tilde{\omega }}_{f}^{T}{\dot{\hat{\omega }}}_{f}+\frac{1}{\lambda_{2}}{\tilde{v}}_{dis}^{T}{\dot{\hat{v}}}_{dis}\\ =s^{T}\left[-\varepsilon_{f}-\varepsilon_{dis}-\eta \mathrm{sgn}\left(s\right)\right]+{\tilde{\omega }}_{f}^{T}\left[s^{T}h_{f}\left(x\right)+\frac{1}{\lambda_{1}}{\dot{\hat{\omega }}}_{f}\right]+{\tilde{v}}_{dis}^{T}\left[s^{T}h_{dis}\left(x\right)+\frac{1}{\lambda_{2}}{\dot{\hat{v}}}_{dis}\right] \end{array}$$

The design of the adaptive rate is the following:

(42) $${\dot{\hat{\omega }}}_{f}=-\lambda_{1}s^{T}h_{f}\left(x\right)$$

(43) $${\dot{\hat{v}}}_{dis}=-\lambda_{2}s^{T}h_{dis}\left(x\right)$$

Substituting Equation (42) and Equation (43) into Equation (41), the following result can be obtained:

(44) $$\begin{array}{c} \dot{L}=s^{T}\left(-\varepsilon_{f}-\varepsilon_{dis}-\eta \mathrm{sgn}\left(s\right)\right)\\ =s^{T}\left(-\varepsilon_{f}-\varepsilon_{dis}\right)-\eta \left|s\right| \end{array}$$

The approximation errors $\varepsilon_{f}$ and $\varepsilon_{dis}$ of the neural network are positive real numbers that approach zero. Setting $\eta \geq \left|\varepsilon_{f}+\varepsilon_{dis}\right|$, we obtain $\dot{L}\leq 0$. When $\dot{L}\equiv 0$, s=0, and according to LaSalle’s invariance principle, the closed-loop system exhibits asymptotically stability. When t→∞, thus $e\to 0,\;\dot{e}\to 0$.

**Figure 9:**
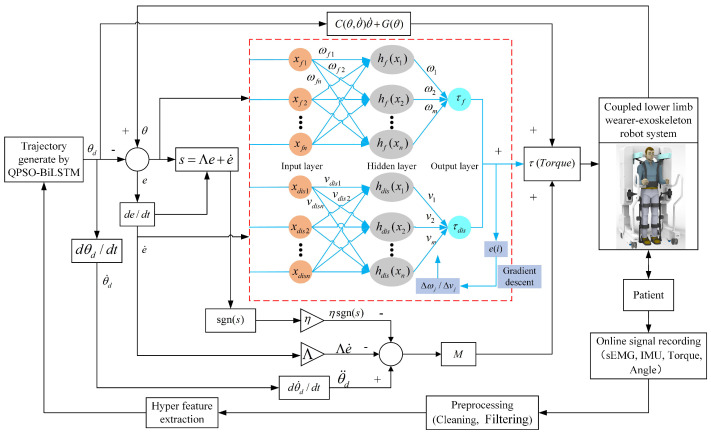
DRBFNNASMC controller structure.

The designed DRBFNNASMC controller is illustrated in Figure 9. The inputs to this controller consist of the discrepancy between the target and actual angle values *e*, as well as the derivative of this error, represented as $\dot{e}$. The output is the motor torque τ. The motor torque τ acts on the coupled lower-limb wearer-exoskeleton robotic system, facilitating rehabilitation training for the patient. By recording sEMG, IMU, and motor encoder torque and angle data in real-time, feature data are extracted, and the QPSO-BiLSTM algorithm predicts trajectories to generate the patient’s required gait trajectory. By further integrating the dual RBF neural network with sliding mode control, the DRBFNNASMC controller is proposed to track the gait trajectory predicted by the QPSO-BiLSTM. Ultimately, the stability of the system is established through the application of the Lyapunov stability theory.

## 3. Results

To validate the effectiveness and accuracy of the proposed control scheme, a series of validation experiments were designed using a follow-up LEERR platform and its related systems, incorporating multiple submodules. The experimental results were then analyzed. Finally, through walking experiments with four participants, the subsystems were integrated to evaluate the overall control performance.

### 3.1. Data Collection and Preprocessing

**Figure 10:**
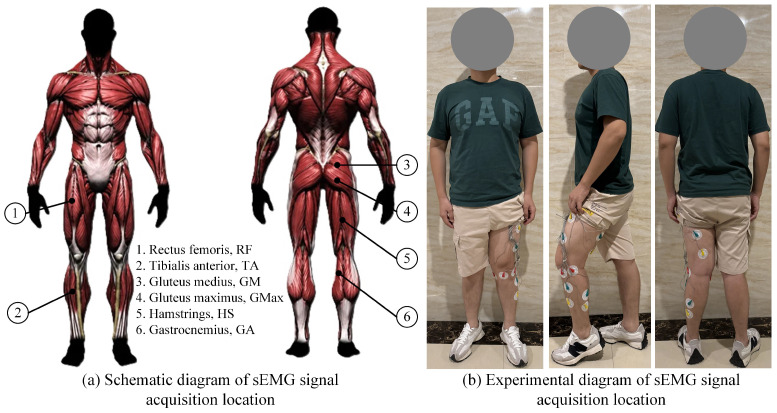
Attachment positions of the six-channel sEMG sensors on the human body.

In the experiment, sEMG signals, joint angles, and binocular vision data were primarily collected. The sEMG signals were acquired using a six-channel sEMG muscle electrical sensor module developed with Arduino, with a sampling rate of 600 kHz. The joint angles were calculated using the WitMotion IMU inertial navigation module with a sampling rate of 200 Hz. To eliminate high-frequency interference, a moving average filter was applied to both types of signals. Figure 10a,b show the schematic diagram and experimental setup of the six-channel sEMG signal acquisition positions, respectively, used to record the flexion/extension movement of the lower limb joints in the sagittal plane.

To process the sEMG signals, a Butterworth filter was applied. Considering the temporal mismatch between the high sampling rate of the sEMG signals and the low sampling rate of the posture angles, downsampling of the sEMG signals is required. The specific implementation is as follows:

(45) $$sEMG_{processed}\left(n\right)=\frac{1}{M}\sum_{K=0}^{M-1}sEMG_{original}\left(nM+k\right)$$

where $sEMG_{processed}$ is the downsampled sEMG sequence, $sEMG_{original}$ is the original sEMG sequence, *n* is the resampled data points, and *M* is the downsampling rate. To effectively extract the feature correspondence between the sEMG signal and joint angles, and to prevent the features from being overwhelmed by large volumes of data, both signals are normalized to express them in a dimensionless form. For visual information extraction, the chessboard method was used to calibrate the ZED stereo camera, obtaining the camera’s intrinsic, extrinsic, and distortion parameters for image correction.

### 3.2. Follow-Up Tracking Control Experiment

**Figure 11:**
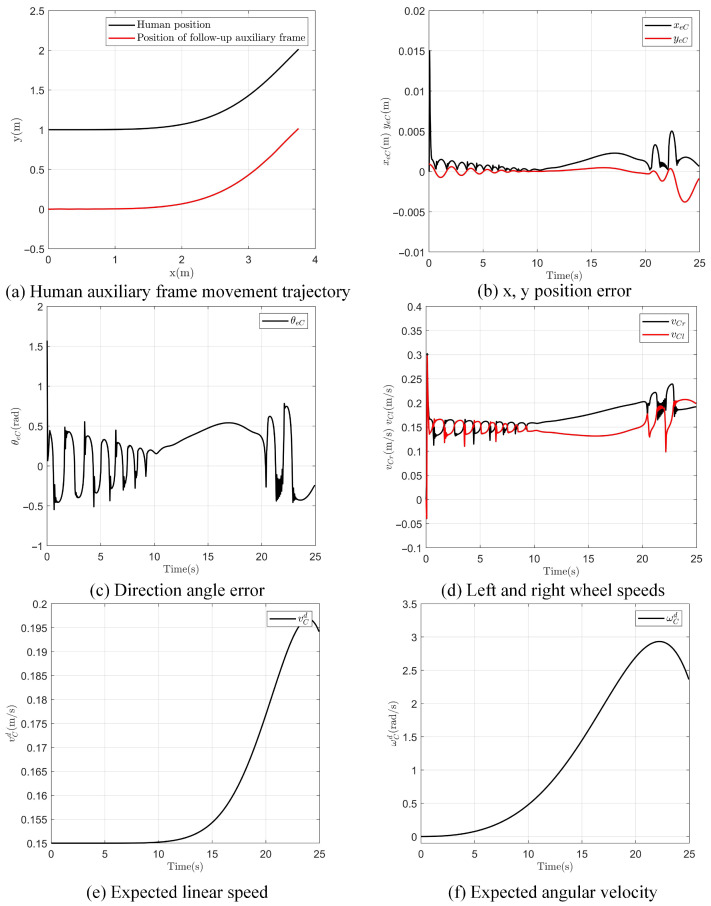
Follow-up assistive frame and human follow-up tracking experiment results.

To verify and assess the viability of the follow-up tracking control, a test experiment was conducted. In the experiment, the follow-up assistive frame, driven by vision-based control, generated control inputs for the two wheels using Equation (11) to achieve follow-up tracking of human walking. During the experiment, the controller’s control parameters were set as follows: $k_{1}=10,\;k_{2}=0.1,\;k_{3}=0.5,\;$$l^{d}=1,\;\psi^{d}=\pi \;/\;$2, and d = 0.4. The findings from the experiment are presented in Figure 11.

Figure 11a shows the movement trajectory of a person and the follow-up assistive frame in the *x*-*y* plane. Under the initial conditions, the person is 1 m in front of the follow-up assistive frame, which continuously maintains a following distance of 1 m. Figure 11b displays the position error in the *x* and *y* directions, with the results showing that the error is bounded, remaining between −0.005 m and 0.015 m. Figure 11c shows the directional angle error between the person and the follow-up assistive frame, with the error range between −0.6° and 1.6°. Figure 11d presents the left and right wheel speeds of the follow-up assistive frame, calculated using Equation (5) to control the frame’s movement. Figure 11e,f show the desired linear and angular velocities of the follow-up assistive frame, respectively, which are determined by the person’s posture relative to the frame.

From the above analysis, it is demonstrated that the follow-up assistive frame can achieve trajectory tracking of a person in the plane under vision-driven control. It should be noted that when the follow-up assistive frame is used in conjunction with the exoskeleton, since the exoskeleton only performs flexion/extension movements in the sagittal plane, the motion of the follow-up assistive frame is simplified to linear tracking in the forward and backward directions.

### 3.3. QPSO-BiLSTM Network Training and Gait Prediction

To build the training dataset for the QPSO-BiLSTM network, four participants (age: 24 ± 4 years, height: 168 ± 10 cm, weight: 72 ± 8 kg) were invited to participate in the experiment. After becoming fully familiar with the experimental procedures, the participants wore six-channel sEMG sensors, as illustrated in Figure 10, with sensors affixed to six muscles of the lower extremities to collect sEMG signals. Meanwhile, an IMU sensor was attached to the participant’s torso to collect posture angle signals. Combining these two types of signals, the angles of the lower limb joints in the sagittal plane were obtained.

During data collection, participants walked back and forth 15 times at a normal walking speed, with each walk lasting 2 min, followed by a 1-min break. During walking, each participant’s sEMG signals and joint angle signals were collected in real time to construct the training dataset. Since each participant has individualized muscle characteristics and joint motion models, a personalized continuous motion intention recognition model was trained for each participant. After processing the signals collected from a single participant, a total of 120,000 data points were generated, with six-channel sEMG signals as inputs and two-channel joint angles as outputs for training the QPSO-BiLSTM network. The entire input dataset was partitioned into two segments, with 90% allocated for model training and the remaining 10% designated for model testing. The network training was conducted using the MATLAB Deep Learning Toolbox, with model parameters set as illustrated in Table 1.

**Figure 12:**
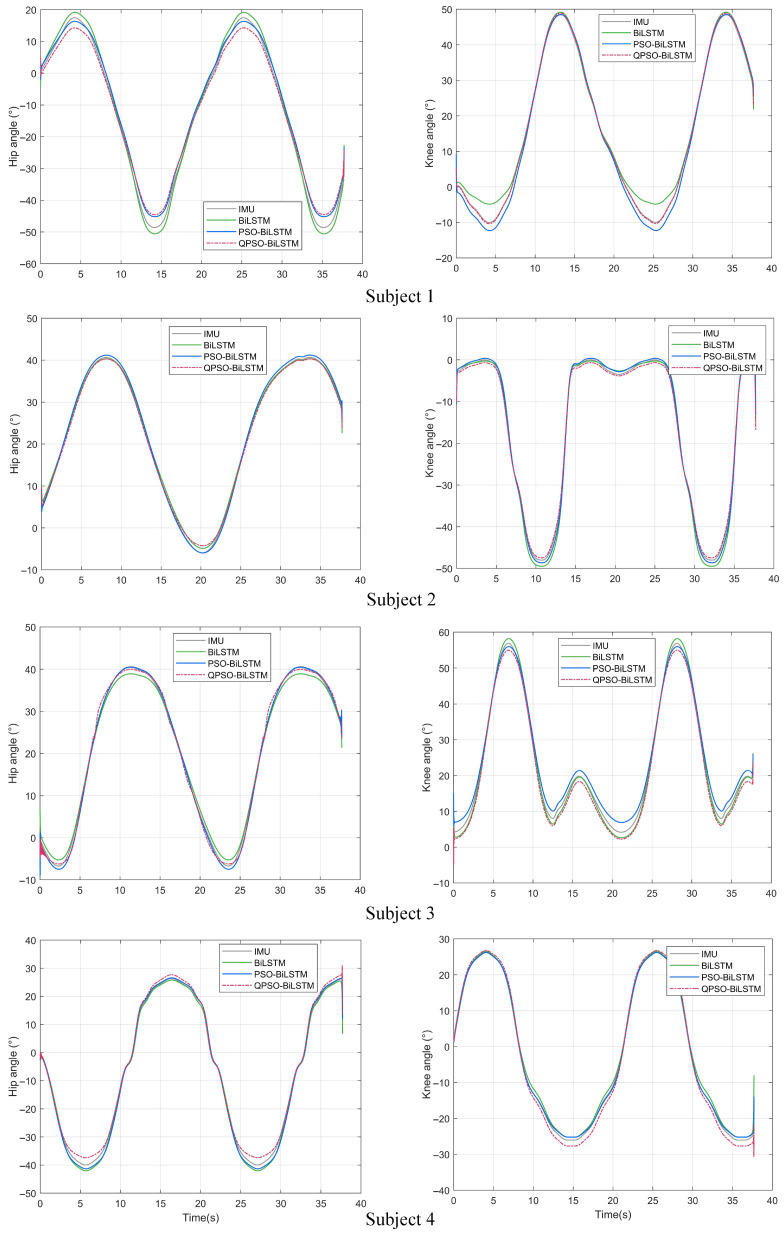
Comparison of joint angle predictions by three network models and IMU measured values. (IMU is an abbreviation for inertial measurement unit, BiLSTM stands for bi-directional long short-term memory network, PSO-BiLSTM denotes particle swarm optimization bi-directional long short-term memory network, and QPSO-BiLSTM refers to quantum-behaved particle swarm optimization bi-directional long short-term memory network).

To evaluate the predictive performance of the QPSO-BiLSTM network, a comparative experiment was conducted. In the experiment, the participants walked at a normal speed at random, with their sEMG signals and joint angles collected. These signals were processed and used as test data. The test data were input into the trained QPSO-BiLSTM network to obtain the predicted joint angles, referred to as the QPSO-BiLSTM network predictions. The joint angles collected by the IMU sensor served as the true values, referred to as the IMU true values. To further compare the recognition performance, additional comparative experiments were conducted using the BiLSTM network and the PSO-BiLSTM network, with their respective predictions referred to as the BiLSTM network predictions and the PSO-BiLSTM network predictions. The findings of the experiment are presented in Figure 12.

To quantitatively analyze prediction accuracy, Equtions (23)–(26) were used to evaluate four aspects: $\delta_{MAE}$, $\delta_{MAPE}$, $\delta_{RMSE}$, and $R^{2}$. The findings are presented in Table 2. The analysis indicates that the predictions of all three models correlate closely with the true values. However, for the four participants, the prediction error associated with the BiLSTM network model was markedly higher than that of the PSO-BiLSTM network, while the QPSO-BiLSTM network exhibited the lowest prediction error. In summary, the QPSO-BiLSTM network can achieve continuous real-time prediction of joint angles and effectively recognize human motion intentions.

### 3.4. LEERR Trajectory Tracking Control Experiment Based on Motion Intention Recognition

**Figure 13:**
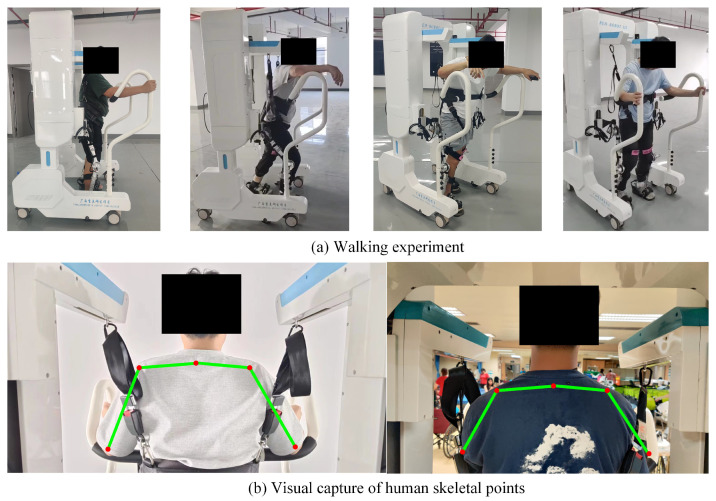
Diagram of the walking experiment. (The green lines represent the main skeleton of the human body, and the red dots represent the main key points of the human body).

To validate the effectiveness of exoskeleton gait control based on motion intention recognition, a walking experiment was designed and conducted using the developed system. Four participants who contributed to BiLSTM network development were invited to participate in this experiment. They wore six-channel sEMG sensors for joint angle prediction. Meanwhile, a vision-driven follow-up assistive frame was used to provide weight support, helping the participants maintain balance while walking. During the experiment, the simulated hemiplegic patients moved their unaffected limb first, while the joint trajectories generated from motion intentions drove the exoskeleton to move the affected limb. The gait phase of both sides was monitored through feedback from foot pressure sensors. To address potential discrepancies between the actual initial position of the affected limb and the desired initial joint trajectory, a linear interpolation method was applied for transition adjustment. Figure 13 shows a schematic of the walking experiment, including the walking experiment setup (a) and the skeletal points captured visually during movement (b).

**Figure 14:**
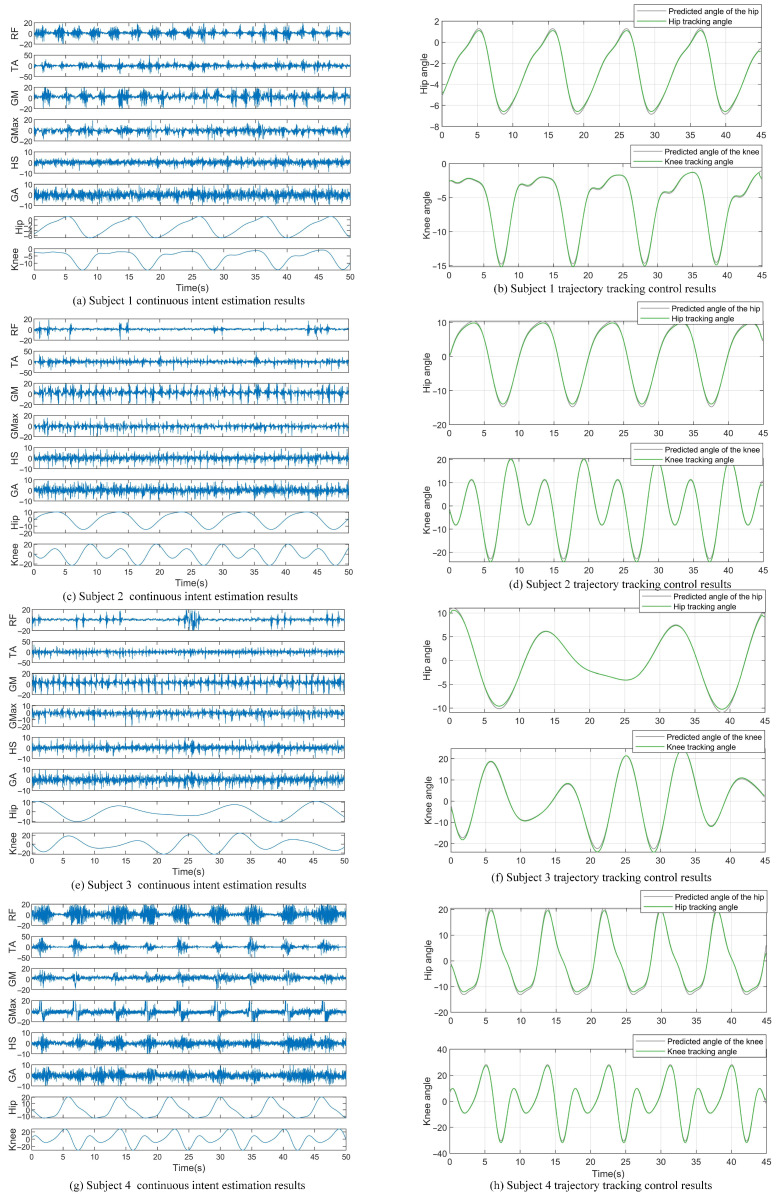
Trajectory tracking control results based on continuous motion intention estimation. RF stands for rectus femoris, TA refers to tibialis anterior, GM denotes gluteus medius, Gmax represents gluteus maximus, HS indicates hamstrings, and GA stands for gastrocnemius.

Figure 14 shows the real-time joint angle predictions and trajectory tracking results of four participants during walking, utilizing six-channel sEMG signals. In the experiment, the controller parameters were set as follows: Λ=10, η=1000b=3, $c=\left[-1\;-0.5\;0\;0.5\;1\right]$, $\lambda_{1}=300,$ and $\lambda_{2}=350,$ with the initial neural network weights set to 0.1. The generated trajectory was predicted in real time by the server to ensure both limbs maintained the same movement speed. Thus, the time reference point on the left graph represents the moment when the prediction side began movement, while the right graph’s time reference is set to when the control side started. The experimental results demonstrate that the trajectory predicted by the multi-channel sEMG signals combined with the QPSO-BiLSTM algorithm exhibited periodic changes, and the controller effectively tracked the generated gait trajectory. This indicates that the scheme of utilizing continuous motion intention recognition to enable the unaffected side to drive the affected side is feasible. Specifically, once the algorithm detects the movement intention, the subjects will be instructed to allow the robot to move autonomously, thereby guiding the movement of the affected limb. To minimize any inadvertent assistance, we suggest that future studies include trials where the recognition algorithm fails to assess the effect of voluntary muscle intention. Additionally, we will consider recruiting naive subjects who are unfamiliar with the study’s objectives to reduce potential bias.

**Figure 15:**
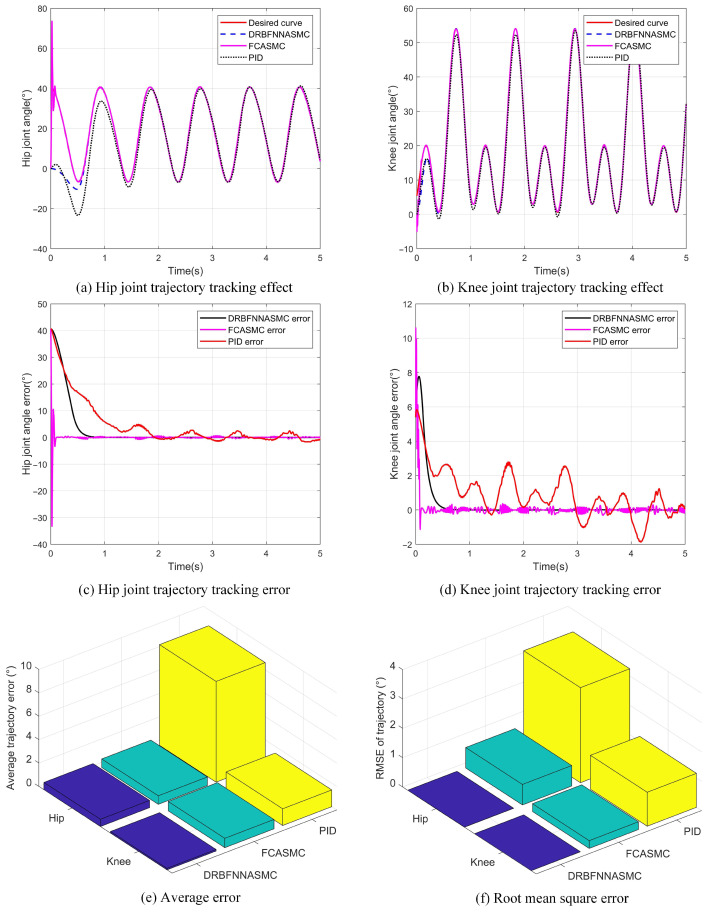
Comparison of the control effectiveness of three different controllers. (DRBFNNASMC stands for dual radial basis function neural network adaptive sliding mode controller, FCASMC refers to fuzzy compensated adaptive sliding mode controller, and PID denotes proportional–integral–derivative controller).

To evaluate the performance of the designed DRBFNNASMC controller, we designed and conducted a comparative experiment. The experiment was divided into three groups, employing the DRBFNNASMC trajectory tracking controller designed in this study, the FCASMC trajectory tracking controller, and the traditional PID trajectory tracking controller. In the experiment, the exoskeleton was used to track a segment of the gait trajectory, and the experimental results are illustrated in Figure 15.

Figure 15a illustrates the tracking performance of the three controllers on the hip joint trajectory. As indicated by the black dashed line, the PID controller demonstrated significant error in the initial 2 s of the gait cycle and was unable to effectively respond to external disturbances, resulting in motor instability and failure to accurately track the desired gait trajectory. In contrast, the DRBFNNASMC and FCASMC controllers produced tracking errors only within the first 0.5 s and then stably tracked the desired gait trajectory. Figure 15b shows the tracking performance of the three controllers on the knee joint trajectory. As indicated by the black dashed line, the PID controller displayed significant oscillations throughout the gait cycle, with errors failing to converge. In comparison, the DRBFNNASMC and FCASMC controllers achieved sliding mode stability, with error convergence and stable tracking of the desired gait trajectory. Overall, the DRBFNNASMC and FCASMC controllers significantly outperform the PID controller in trajectory tracking.

Furthermore, Figure 15c,d show the display of the error curves for the hip and knee joints. The FCASMC controller’s error curve converged to zero within 0.2 s but then displayed periodic oscillations, failing to fully eliminate the effects of external disturbances and friction. This sustained high-frequency oscillation could adversely affect the motor and potentially lead to motor damage.

In conclusion, the trajectory tracking performance of the three controllers was evaluated based on average error and root mean square error (RMSE). Figure 15e shows the average tracking errors for the hip and knee joints under the three control schemes. The PID controller’s average errors for the hip and knee joints were 8.61° and 1.43°, respectively; for the FCASMC controller, they were 0.73° and 0.8°; and for the DRBFNNASMC controller, they were 0.62° and 0.13°. Specifically, the DRBFNNASMC controller’s average error for the hip joint was reduced by 15.1% and 92.8% compared to the FCASMC and PID controllers, respectively, while the average error for the knee joint was reduced by 83.75% and 90.9%. Figure 15f shows the RMSE for the hip and knee joint trajectory tracking under each control scheme. The RMSE values for the PID controller’s hip and knee joints were 3.24° and 1.157°, respectively; for the FCASMC controller, 0.699° and 0.265°; and for the DRBFNNASMC controller, 0.0014° and 0.000276°. Specifically, the DRBFNNASMC controller’s hip joint RMSE was reduced by 99.8% and 99.9% compared to the FCASMC and PID controllers, respectively, while the knee joint RMSE was reduced by 99.9% and 100%. In summary, the DRBFNNASMC controller demonstrated superior trajectory tracking capability in the exoskeleton robot system under external disturbances and friction.

## 4. Discussion

The vision-driven follow-up tracking control strategy can capture and respond to human motion in real time, ensuring that the rehabilitation robot’s movement trajectory closely aligns with the natural motion trajectory of the human body. This is critical for the effectiveness of rehabilitation training, as precise motion trajectory tracking can reduce unnatural or uncoordinated movements during the rehabilitation process, enhancing the effectiveness and safety of the training.

The results in Table 2 show that the proposed QPSO-BiLSTM algorithm successfully decodes the gait trajectories of the hip and knee joints during walking by utilizing QPSO-BiLSTM and sEMG signals to construct a muscle–machine interface. The experimental results further indicate that the BiLSTM network demonstrates significant advantages in multi-channel sEMG signal-based motion intention recognition, particularly in multi-feature and sequential prediction tasks. The application of the QPSO intelligent optimization algorithm further enhances the performance of the BiLSTM network model by optimizing parameters online, addressing shortcomings in real-time performance and adaptability. However, this approach also has some limitations. Currently, our algorithm is trained based on gait data from healthy individuals, and therefore may not accurately capture the gait intentions of stroke patients. To address this limitation, we plan to collect more sEMG data from stroke patients in future studies and optimize the existing algorithm for these data. We will also explore personalized training methods, adjusting the algorithm according to the specific conditions of the patients to improve its accuracy and applicability across different patient types. To better assess the algorithm’s applicability to stroke patients, future research will include diversified testing on different groups of stroke patients and attempt to combine other physiological signals (such as motion sensor data) to further enhance the accuracy of gait intention detection.

The average error and RMSE results in Figure 15e,f indicate that the DRBFNNASMC control algorithm significantly outperforms the FCASMC control algorithm and the traditional PID control algorithm in terms of error metrics. This demonstrates that the DRBFNNASMC control algorithm offers higher accuracy and stability in trajectory tracking.

Despite achieving significant results in several areas, there remain some issues that require further exploration. First, the generalization ability of the QPSO-BiLSTM model across different participants and movement tasks needs further validation. Second, the computational complexity and real-time performance of the DRBFNNASMC controller in complex nonlinear systems require further optimization. Additionally, the long-term effects and patient satisfaction with the follow-up assistive frame and rehabilitation robot system in practical clinical applications need further clinical validation.

## 5. Conclusions

This study proposes an active control method for a follow-up LEERR based on human motion intention recognition, and multiple experiments have verified its significant advantages. Initially, the experimental results indicate that the vision-driven follow-up tracking control strategy effectively supports body weight during walking, compensates for the vertical movement of the body’s center of mass, and achieves accurate tracking of the human movement trajectory. Additionally, by constructing a BiLSTM network and applying QPSO algorithm, the QPSO-BiLSTM combined model demonstrates outstanding performance in decoding multi-channel sEMG signals to predict lower limb joint angles. Compared to traditional BiLSTM and PSO-BiLSTM networks, the QPSO-BiLSTM network shows significant improvement in continuous motion prediction of the lower limbs. Furthermore, the designed DRBFNNASMC controller generates precise control torques to effectively drive the robot in tracking the trajectory generated by the muscle–machine interface, even in the face of uncertain nonlinear dynamics of the wearer-exoskeleton system. The experimental results show that the DRBFNNASMC controller outperforms the FCASMC control algorithm and traditional PID control algorithm in gait tracking. The proposed method holds great potential for clinical rehabilitation applications, providing a more intelligent, precise, and efficient solution for lower limb rehabilitation training. It enhances the intelligence level of the LEERR, achieving breakthroughs in trajectory tracking and motion intention recognition accuracy, and laying a foundation for future research and application of rehabilitation robots. Future research will continue to optimize and validate this method to promote its practical application in a broader range of rehabilitation scenarios.

## Figures and Tables

**Table 1:** QPSO-BiLSTM network model training parameter settings.

Model Solver	Maximum Number of Iterations	Gradient Threshold	Initial Learning Rate	Learning Rate Reduction Cycle	Learning Rate Decline Factor
Adam optimizer	88	1	0.005	65	0.1

**Table 2:** Quantitative analysis of joint angle predictions by three networks ($\delta_{MAE}$ is an abbreviation for mean absolute error, $\delta_{MAPE}$ denotes mean absolute percentage error, $\delta_{RMSE}$ stands for root mean square error, and $R^{2}$ refers to coefficient of determination).

		Hip			Knee				
		$\delta_{\mathrm{MAE}}$	$\delta_{\mathrm{MAPE}}$	$\delta_{\mathrm{RMSE}}$	$R^{2}$	$\delta_{\mathrm{MAE}}$	$\delta_{\mathrm{MAPE}}$	$\delta_{\mathrm{RMSE}}$	$R^{2}$
Subject1	BiLSTM	0.5803	58.86%	0.7689	0.9468	0.5155	12.06%	0.8099	0.9786
	PSO-BiLSTM	0.5204	46.95%	0.6542	0.9615	0.6916	55.46%	0.9583	0.9701
	QPSO-BiLSTM	0.3152	16.79%	0.3891	0.9864	0.1564	4.36%	0.2806	0.9974
Subject2	BiLSTM	0.5606	39.35%	0.8904	0.9699	0.2238	36.22%	0.2955	0.9810
	PSO-BiLSTM	0.7565	43.59%	0.9015	0.9691	0.1599	35.98%	0.2123	0.9902
	QPSO-BiLSTM	0.4759	38.35%	0.7233	0.9801	0.0495	8.15%	0.0808	0.9986
Subject3	BiLSTM	1.7713	53.32%	2.4841	0.9785	0.2668	21.44%	0.3516	0.9890
	PSO-BiLSTM	1.7151	37.20%	2.5195	0.9779	0.3621	27.00%	0.4260	0.9839
	QPSO-BiLSTM	0.9908	33.64%	1.1992	0.9950	0.1810	8.82%	0.2492	0.9945
Subject4	BiLSTM	0.4783	1.78%	0.7041	0.9684	0.4709	6.28%	0.6583	0.9048
	PSO-BiLSTM	0.3645	1.44%	0.4820	0.9852	0.4444	6.13%	0.5679	0.9291
	QPSO-BiLSTM	0.2251	0.87%	0.3595	0.9920	0.4643	6.38%	0.5447	0.9348

## Data Availability

Data are contained within the article.
